# Supplementary material for: Reduced FOXF1 links unrepaired DNA damage to pulmonary arterial hypertension
Source: Nat Commun. 2023 Nov 21;14:7578. doi: 10.1038/s41467-023-43039-y (PMC10663616; doi:10.1038/s41467-023-43039-y)
Supplement: Supplementary file 1 — Supplementary Information [file 41467_2023_43039_MOESM1_ESM.pdf]

Supplementary Information

Supplementary Table1: EC-*Atm*$^{-/-}$ mouse blood sample data

	**Normoxia**		**Hypoxia**		**Reoxygenation**	
	**Con**	**EC-*Atm*$^{-/-}$**	**Con**	**EC-*Atm*$^{-/-}$**	**Con**	**EC-*Atm*$^{-/-}$**
WBC (x10^3)/µl	4.31 ± 0.77	4.40 ±0.61	4.22 ± 0.39	4.06 ± 0.62	3.36 ± 0.73	4.23 ± 1.35
RBC (x10^6)/µl	9.78 ± 0.13	9.14 ± 0.17	11.85± 0.35	11.56± 0.31	9.54 ± 0.48	8.74 ± 0.56
Hb (gm/dl)	14.43 ± 0.24	12.97 ± 0.14	17.80 ± 0.83	17.43 ±0.44	13.27 ± 0.62	12.76 ± 0.59
Hct (%)	45.13 ± 1.00	40.37 ± 0.35	56.20 ± 1.93	53.77 ± 1.41	41.53 ± 0.98	38.77 ± 1.92
Plt (x10^6)/µl	1.23 ± 0.16	1.25 ± 0.24	0.89 ± 0.11	1.09 ± 0.14	1.47 ± 0.19	1.38 ± 0.31
Reticulocyte (%)	2.77 ± 0.09	4.68 ± 0.73	1.79 ± 0.21	2.51 ± 0.25	4.19 ± 0.33	5.23 ± 0.86
Neutrophils (%)	23.67 ± 4.63	44.67± 7.06	21.67 ± 2.19	24.67 ± 1.45	53.00 ± 7.64	40.00 ± 2.00
Lymphocytes (%)	69.00 ± 5.03	45.67 ± 8.95	65.33 ± 2.67	66.00 ± 2.31	40.33 ± 6.39	52.00 ± 4.00
Monocytes (%)	5.00 ± 2.52	9.00 ± 2.08	9.00 ± 1.00	7.33 ± 0.88	3.00 ± 0.58	5.00 ± 1.00
Eosinophils (%)	2.00 ± 0.58	0.67 ± 0.67	4.00 ± 1.15	2.00 ± 0.00	3.66 ± 1.45	3.00 ± 1.73

Data are mean ± SEM, of n=3 male mice in each group. There is no significant difference between control and EC-*Atm*$^{-/-}$ mice under the same conditions by 2-sided Unpaired t-test. Multiple comparisons were not applied.

Supplementary Table 2: Top 50 upregulated or downregulated genes based on Padj.

The top 50 Padj (adjusted p-value) genes in EC-*Atm*$^{-/-}$ or EC-*Bmpr2*$^{-/-}$ mice following reoxygenation, vs. control mice under reoxygenation. Upregulated genes in the left column, downregulated genes on the right. 2-sided Wald test was performed. p-values were adjusted to get Padj using the Benjamini-Hochberg method. reoxy: reoxygenation

**a. EC-*Atm*$^{-/-}$ vs. control**	
**Upregulated in EC-*Atm*$^{-/-}$ reoxy vs. control reoxy**	**Downregulated in EC-*Atm*$^{-/-}$ reoxy vs. control reoxy**
Sparcl1	*Casc4*
Sparc	*Adgrf5*
Col4a1	*Cyth3*
Fabp4	*Ablim1*
Col4a2	*Ankrd33b*
Scarb1	*Sox11*
Anxa2	*Rnf144a*
Chil1	*Adamts1*
H2-T24	*Tmem100*
Cdh13	*Ece1*
Cd34	*Vegfr2 (Kdr)*
Ntrk2	*Tmod2*
Gda	*Ehd4*
Trp53i11	*Arhgef12*
Slc6a6	*Ednrb*
Itih5	*Klf4*
Eln	*Mndal*
Hspg2	*Pydc3*
Ucp2	*Cyp4b1*
Pde2a	*Emp2*
Lrg1	*Fmo1*
Timp3	*Ripply3*
Col15a1	*Fgf1*
	Klf2
	Abi3bp
	Foxf1
	S1pr1

**b. EC-*Bmpr2*$^{-/-}$ vs. control**	
**Upregulated in EC *Bmpr2-/-* reoxy vs. control reoxy**	**Downregulated in EC *Bmpr2-/-* reoxy vs. to control reoxy**
Lyve1	*Acvrl1*
Chil3	*Hpgd*
Mpeg1	*Ankrd33b*
Ccl6	*Casc4*
Rn45s	*Plxna1*
Ctss	*Cyyr1*
Cybb	*Limch1*
Jchain	*Gpx3*
Lyz2	*Nrp1*
Mrc1	*Cd93*
Ahnak	*Clec14a*
Trp53i11	*Fmo1*
Ivns1abp	*Mfap4*
Npr3	*Podxl*
Itgb2	*Arhgef12*
Cd44	*Adgre5*
Ptprc	*Cxcl12*
Bhlhe40	*Tmem100*
	Pcdh1
	Adgrf5
	Pdgfra
	Smad6
	Eng
	Gsn
	Vegfr2 (Kdr)
	Tbx3
	Itpripl2
	Tspan18
	Myzap
	Hbb-bs
	Sema3c
	Bmpr2

Supplementary Table 3: Demographics of donors and PAH patients (lung samples)

a. Donor Controls:

Average age: 41.2 yrs (Range: 28 - 54 yrs; Median: 43 yrs)

Donor	Gender	Experiment
1	F	IF
2	M	IF
3	M	IF, Cell PCR
4	F	IF
5	M	Cell PCR
6	F	Cell PCR
7	M	Cell PCR
8	M	Cell PCR
9	F	Cell PCR

b. PAH Patients

Average age: 35.2 yrs (Range: 23 – 56 yrs; Median: 33 yrs)

Patient	Gender	Experiment	Diagnosis[1]	PAP[2] (s/d/m)	PVR[3] (WU)	6MWD[4] (m)	BMPR2 mutation	Medications
1	F	IF, cell PCR	FPAH	87/29/48	9.74	288	Nonsense; Exon7(c961C>T, pR321X)	bosentan, treprostinil, sildenafil, epoprostenol
2	F	IF	FPAH	110/65/82	16.04	375.8	Splice site; Intron6(c853-2A>G)	sildenafil, ambrisentan, tadalafil, treprostinil
3	F	IF, cell OE (PAH3 in Fig. 5)	FPAH	75/33/48	15.57	326.1	Missense; Exon11 (c1471C>T, pR491W)	epoprostenol, bosentan, sildenafil, treprostinil
4	F	IF	IPAH	84/40/58	17.98	292.6	Missense; Exon11 (c1450T>C, pW484R)	bosentan, iloprost, epoprostenol
5	M	Cell OE (PAH1 in Fig. 5)	FPAH	119/51/77	14.22	309	Missense; Exon11 (c1471C>T, pR491W)	sildenafil, sitaxsentan, ambrisentan, epoprostenol, treprostinil
6	F	Cell OE (PAH2 in Fig. 5), cell PCR	IPAH	110/49/69	12.11	359.7	c.76+5G>GA probable mutation, not in dbSNP; may disrupt splicing	sildenafil, treprostinil, bosentan, iloprost

Patient	Gender	Experiment	Diagnosis[1]	PAP[2] (s/d/m)	PVR[3] (WU)	6MWD[4] (m)	BMPR2 mutation	Medications
7	F	Cell PCR	IPAH	96/47/66	11.99	368	Splice site; Intron7(c966A> T, p.G322G)	sildenafil, epoprostenol
8	F	Cell PCR	IPAH	83/39/57	11.41	137.2	none	sildenafil, ambrisentan, IV treprostinil
9	F	Cell PCR	IPAH	75/43/55	9.84	472.4	none	sildenafil, bosentan, IV epoprostenol
10	M	Cell PCR	IPAH	90/51/68	11.38	423.7	none	bosentan, sildenafil, epoprostenol
11	F	IF	IPAH	76/40/47	-	161	none	IV epoprostenol, bosentan, ambrisentan, sildenafil
12	F	IF	IPAH	84/26/47	-	294	none	ambrisentan, sildenafil, inhaled iloprost, IV epoprostenol
13	M	IF	IPAH	73/29/48	9.56	356	none	sildenafil, ambrisentan, IV epoprostenol, bosentan
14	M	IF	IPAH	65/15/36	-	510.5	none	IV epoprostenol, sildenafil, IV treprostinil

[1] Diagnosis: FPAH, Familial PAH; IPAH, Idiopathic PAH

[2] PAP: Pulmonary artery pressure (mmHg), s: systolic, d: diastolic, m: mean.

[3] PVR: pulmonary vascular resistance in Wood Units (WU)

[4] 6MWD: distance (m) walked in 6 minutes

Supplementary Table 4: RT-PCR Primers

Human	Forward	Reverse
BMPR2	TCTGGATCTTTCAGCCACAA	CCTGATCCTGATTTGCCATC
ATM	ATCTGCTGCCGTCAACTAGAA	GATCTCGAATCAGGCGCTTAAA
FOXF1	CCCAGCATGTGTGACCGAAA	ATCACGCAAGGCTTGATGTCT
CLDN5	CTCTGCTGGTTCGCCAACAT	CAGCTCGTACTTCTGCGACA
CD31	CCAAGGTGGGATCGTGAGG	TCGGAAGGATAAAACGCGGTC
VEGFR2	GGCCCAATAATCAGAGTGGCA	CCAGTGTCATTTCCGATCACTTT
TP53	CAGCACATGACGGAGGTTGT	TCATCCAAATACTCCACACGC
GAPDH	AGCCACATCGCTCAGACAC	TTAAAAGCAGCCCTGGTGAC
β-ACTIN	AGAGCTACGAGCTGCCTGAC	AGCACTGTGTTGGCGTACAG

Mouse	Forward	Reverse
Bmpr2	GACAGGAGACCGGAAACAGG	TATCCAGGTCAAGGGAGGGC
Atm	AGGCCAAATGATTTCAGTGC	TGCGTGTATATGCCAATCGT
Foxf1	ACCAAAACAGTCACAACGGG	GCTGGTGATAGTAAGATCCTCCG
Gapdh	TGACCTCAACTACATGGTCTACA	CTTCCCATTCTCGGCCTTG
β-Actin	GTGACGTTGACATCCGTAAAGA	GCCGGACTCATCGTACTCC
Cd31	ACCGGGTGCTGTTCTATAAGG	TCACCTCGTACTCAATCGTGG
Vegfr2	CAAACCTCAATGTGTCTCTTTGC	AGAGTAAAGCCTATCTCGCTGT
Cldn5	TAAGGCACGGGTAGCACTCA	GGACAACGATGTTGGCGAAC

Supplementary Table 5: Antibodies used for western immunoblotting

Antibody	Source	Identifier	Lot	Dilution
BMPR2 (Clone 18)	BD Bioscience	Cat#: 612292	7299907/ 7341692	1:250
Phospho-RPA(S4/S8)	Bethyl	Cat#: A300-245A	N/A	1:1000
RPA	Bethyl	Cat#: A300-244A	N/A	1:1000
γH2AX (Clone JBW 301)	Millpore	Cat#: 05-636	33380399	1:1000
Bactin (Clone C4)	Santa cruz	Cat#: sc-47778	J1113	1:2000
tdTomato	Origene	Cat#: TA150129	0136	1:5000
ATM (Clone D2E2)	Cell Signaling	Cat#: 2873	5	1:1000
VEGFR2 (Clone 55B11)	Cell Signaling	Cat#: 2479	18	1:1000
GAPDH (Clone 14C10)	Cell Signaling	Cat#: 2118	10	1:2000
P53 (Clone DO-1)	Santa Cruz	Cat#: sc-126	J0113	1:500
FOXF1	R and D	Cat#: AF4798	AHH0420091	1:1000
FOXF1	Abcam	Cat#: ab168383	GR3399493-4	1:1000
CLDN5 (Clone 4C3C2)	ThermoFisher	Cat#: 35-2500	359666A	1:500
DNA-PK	Cell Signaling	Cat#: 4602T	2	1:1000
ATR	Cell Signaling	Cat#: 2790S	10	1:1000
Anti-mouse horseradish peroxidase (HRP) antibody	Santa cruz	Cat#: sc-2005	H2213	1:5000
Anti-goat horseradish peroxidase (HRP) antibody	Santa cruz	Cat#: sc-2953	D2916	1:5000
Anti-rabbit horseradish peroxidase (HRP) antibody	Santa cruz	Cat#: sc-2004	B2216	1:5000
Anti-rabbit horseradish peroxidase (HRP) antibody	Jackson ImmunoResearch	Cat#: 211-032-171	145158	1:5000

Supplementary Table 6: Antibodies used for immunostaining

Antibody	Source	Identifier	Lot	Dilution
αSMA (clone 1A4)	Sigma Aldrich	Cat#: A2547	076M4784V	IF 1:300, IHC 1:400
αSMA	Abcam	Cat#: ab5694	449256	IF 1:50
MECA32	Developmental Studies Hybridoma Bank	Cat#: AB_531797	6/20/19-17ug/ml	IF 1:4
Luciferase	Abcam	Cat#: ab21176	GR3355137-1	IF 1:100
Von Willebrand factor	Abcam	Cat#: ab6994	GR275409-1	IF 1:300
RFP (tdTomato)	Rockland	Cat#: 600-401-379	42872	IF 1:1000
tdTomato	Origene	Cat#: TA150129	0316	IF 1:500
γH2AX	Millpore	Cat#: 05-636	2138016	IF: 1:100, ICC 1:300
Phospho-RPA(S4/S8)	Bethyl	Cat#: A300-245A	N/A	ICC 1:500
Donkey anti-mouse Alexa Fluor 647	Invitrogen	Cat#: A31571	1984047	IF, ICC 1:500
Donkey anti-rabbit Alexa Fluor 647	Invitrogen	Cat#: A31573	1964354	IF, ICC 1:500
Donkey anti-rabbit Alexa Fluor 594	Invitrogen	Cat#: A21207	1890862	IF, ICC 1:500
Donkey anti-goat Alexa Fluor 594	Invitrogen	Cat#: A11058	2045324	IF, ICC 1:500
Donkey anti-mouse Alexa Fluor 488	Invitrogen	Cat#: A21202	1796361	IF, ICC 1:500
Donkey anti-rabbit Alexa Fluor 488	Invitrogen	Cat#: A21206	2045215	IF, ICC 1:500
Donkey anti-goat Alexa Fluor 488	Invitrogen	Cat#: A11055	1869589	IF, ICC 1:500

Supplementary figures

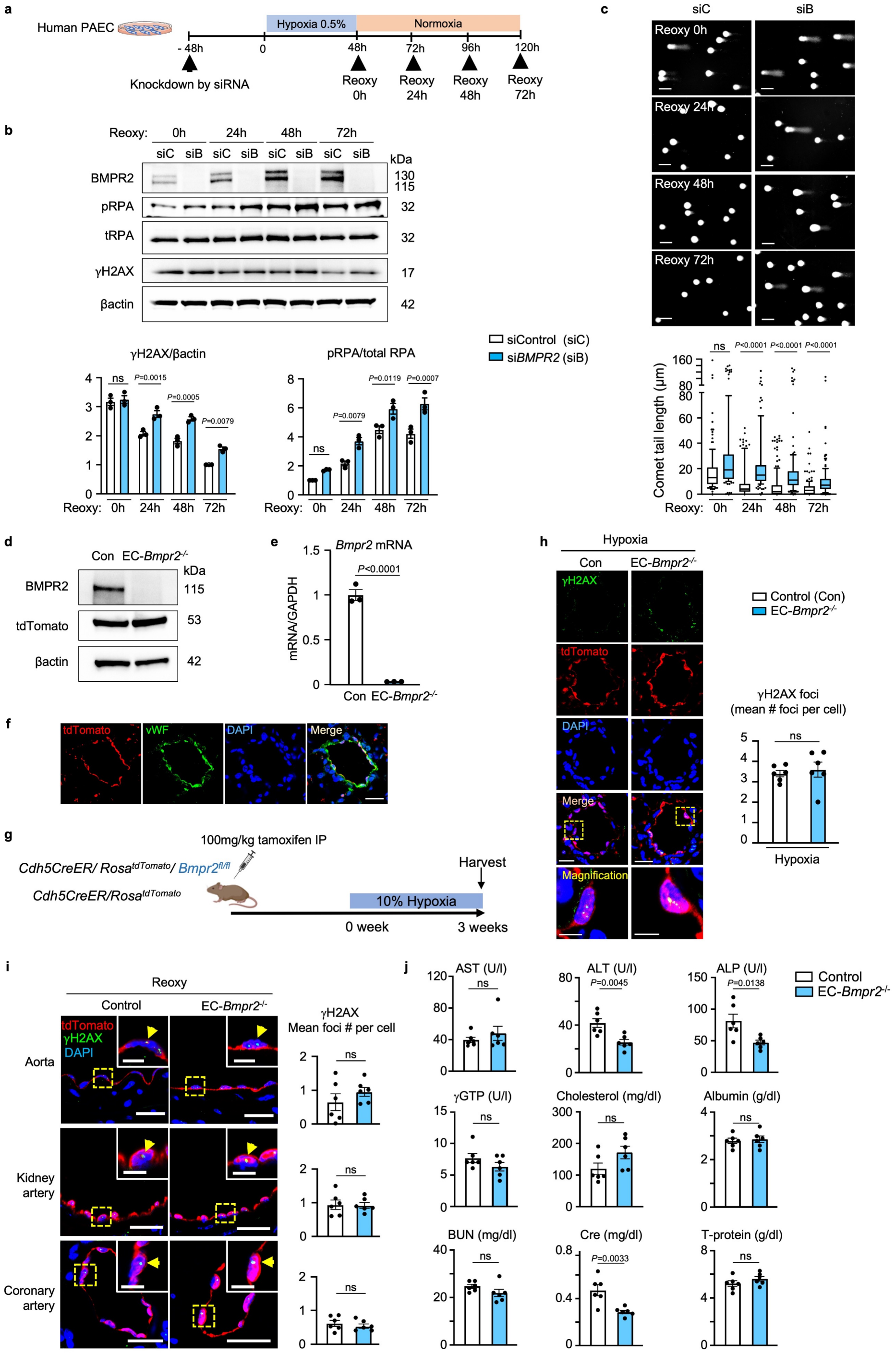

Supplementary Figure 1: Loss of BMPR2 results in accumulated DNA damage during reoxygenation, but not following hypoxia. *(See legend on the next page)*

Supplementary Figure 1: Loss of BMPR2 results in accumulated DNA damage during reoxygenation, but not following hypoxia. a. Schema of experimental design. Cells were harvested 48 hours after hypoxia (Reoxy 0h) and 24, 48, and 72 hours after returning to room air (Reoxy 24, 48, 72h). Schema created with BioRender.com. **b.** Representative immunoblots of the DNA damage markers γH2AX and phosphorylated (p)RPA in human PAEC transfected with scrambled control siRNA (siC) or siRNA targeting *BMPR2* (siB). Cells harvested at Reoxy 0 h were lysed in a hypoxic chamber. Quantification was performed for n=3 individual experiment. Bars represent mean ± S.E.M. P values determined by 2-way ANOVA with Holm-Sidak posthoc test. ns, not significant**. c.** Comet assay showing DNA damage in BMPR2-depleted cells, as reflected by the comet tail length. For measurements at 0h Reoxy, cells were harvested with trypsin in room air immediately upon removal from the hypoxic chamber. Scale bar, 20 µm. Cells n= 137, 118, 115, 108, 177, 135, 160, 151; for siC Reoxy 0h, siB Reoxy 0h, siC Reoxy 24h, siB Reoxy 24h, siC Reoxy 48h, siB Reoxy 48h, siC Reoxy 72h, siB Reoxy 72h, respectively. Box bounds show the 25th and 75th percentiles, whiskers show the 10th to 90th percentiles and the box centre shows the median. P values determined by Kruskal-Wallis ANOVA test with Dunn's test. ns, not significant.

(d-h): *Bmpr2* was deleted specifically in mouse pulmonary EC by injecting tamoxifen in *Cdh5CreER/ Rosa^{tdTomao}/ Bmpr2$^{fl/fl}$* mice (EC-*Bmpr2$^{-/-}$* mice), as described in the Methods. *Cdh5CreER/Rosa^{tdTomato}* were used as controls. **d.** Immunoblot of BMPR2 and tdTomato in pulmonary EC of EC-*Bmpr2$^{-/-}$* and control mice. **e.** mRNA expression of *Bmpr2* in pulmonary EC of EC-*Bmpr2$^{-/-}$* and control mice. n=3 mice per genotype. **f.** Immunohistochemistry indicating tdTomato expression is consistent with the EC marker vWF in EC-*Bmpr2$^{-/-}$* mice after tamoxifen injection. Scale bar, 20 µm. **g.** Schema of experimental design: *Cdh5CreER/ Rosa^{tdTomato}/Bmpr2$^{fl/fl}$* mice (EC-*Bmpr2$^{-/-}$* mice) and *Cdh5CreER/Rosa^{tdTomato}* (control) mice were exposed to hypoxia

(10% oxygen) for 3 weeks after tamoxifen injection. Schema created with BioRender.com.

h. Mean number of γH2AX immunofluorescence foci was measured in PAEC in control and EC-*Bmpr2*$^{-/-}$ mice (arrows). Scale bars, 20 µm. Bottom panels show magnified merged image of the area delineated with the dotted line. Scale bars, 5 µm. n = 6 mice per group. **i,** Mean γH2AX immunofluorescent foci number were measured in five different positions in each aorta, three kidney arteries and five coronary arteries per mouse in control and EC-*Bmpr2*$^{-/-}$ mice after reoxygenation (arrows). Scale bars, 20 µm. Bottom panels show magnified merged image of the area delineated with the dotted line. Scale bars, 5 µm. n =6 mice. **j.** Blood sample from Control and EC-*Bmpr2*$^{-/-}$ mice in reoxygenation. Chemistry reference range[1 2]; AST 35.7-135.7 U/L, ALT 0-228.2 U/L, ALP 60.3- 103.9 U/L, γGTP none, Cholesterol 72.9-123.8 mg/dL, Albumin 2.9-4.5 g/dl, BUN 18-31 mg/dl, Cre (Creatinine) 0.2-1.0 mg/dL, T-protein 4.9-7.1 g/dl.

In (e-j), bars represent mean±S.E.M. P values determined by unpaired 2-sided t-test. ns, not significant. Source data are provided as a Source Data file.

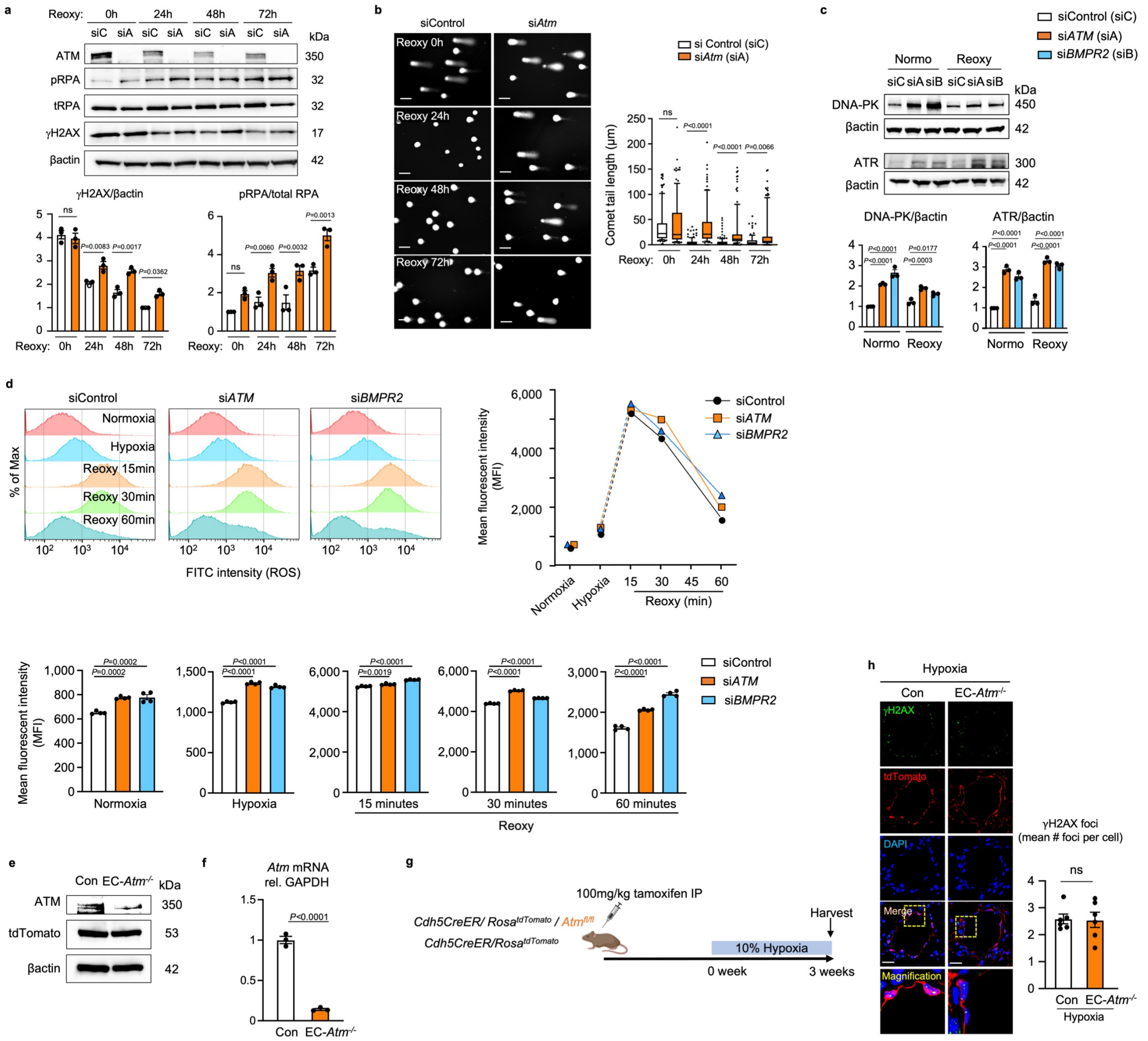

Supplementary Figure 2: Loss of ATM can induce unrepaired DNA damage after hypoxia and during reoxygenation in human PAEC.

ATM was deleted in commercially available human healthy donor PAEC by transfection with siRNA targeting *ATM* (siA) or with scrambled control siRNA (siC). The cells were cultured under hypoxia (0.5% O_2) for 48 h followed by room air for up to 72 h (Reoxy), following the protocol shown in supplementary Figure 1a. **a.** Representative immunoblots of the DNA damage markers γH2AX and phosphorylated (p)RPA. Cells at Reoxy 0h were lysed in a hypoxic chamber. Quantification was performed for n=3 individual experiment. Bars represent mean ± S.E.M. P values determined by 2-way ANOVA with Holm-Sidak posthoc test. ns, not significant**. b.** Comet assay showing DNA damage in ATM-depleted

cells, as reflected by the comet tail length. For the measurement at Reoxy 0h, cells were harvested with trypsin in room air immediately following removal from the hypoxic chamber. Scale bar, 20 µm. Number of Cells: 109, 105, 128, 106, 158, 117, 112, 124, for siC Reoxy 0 h, siB Reoxy 0 h, siC Reoxy 24 h, siB Reoxy 24 h, siC Reoxy 48 h, siB Reoxy 48 h, siC Reoxy 72 h, siB Reoxy 72 h, respectively. Box bounds show the 25th and 75th percentiles, whiskers show the 10th to 90th percentiles and the box centre shows the median. P values determined by Kruskal-Wallis ANOVA test with Dunn's test. ns, not significant**. c**, Representative immunoblots of the DNA damage response kinases, DNA-PK and ATR. Quantification was performed for n=3 for each genotype or condition. Bars represent mean ± S.E.M. P values determined by 2-way ANOVA with Holm-Sidak posthoc test. ns, not significant.

d, ROS production in human healthy donor PAEC following transfection with siRNA targeting *ATM* (siA), *BMPR2* (siB) or scrambled control siRNA (siC) were cultured under normoxia, hypoxia or reoxygenation. ROS was measured by flow cytometry using the highly fluorescent indicator dye DCFDA, with excitation and emission wavelengths of 498 and 522 nm, respectively. Upper left, distribution of ROS fluorescence intensity. Upper right, mean value of mean fluorescence intensity (MFI) during normoxia, after hypoxia, and following reoxygenation for 15 min, 30 min and 60 min. This is visualized as a time course in the upper right panel. The lower bar graphs show the MFI data as Mean ± S.E.M for each experimental condition with statistical analysis of n=4 experiments per condition and P values determined by one way ANOVA with Holm-Sidak posthoc test.

e. Immunoblot of ATM and tdTomato, and **f,** mRNA expression of *Atm* in pulmonary EC of EC-$Atm^{-/-}$ and control mice. Data shown as mean ± S.E.M. P values determined by unpaired 2-sided t-test.

g. Schema of experimental design: *Cdh5CreER/* $Rosa^{tdTomato}$/$Atm^{fl/fl}$ (EC-$Atm^{-/-}$) mice and *Cdh5CreER/*$Rosa^{tdTomato}$ (control) mice were exposed to hypoxia (10% oxygen) for 3 weeks after tamoxifen injection. Schema created with BioRender.com. **h.** γH2AX immunofluorescence foci were measured in PAEC of control and EC-$Atm^{-/-}$ mice. Scale bars, 20 µm. Bottom panel show magnified merged image of the area delineated with the dotted line. Scale bars, 5 µm. n = 6 mice. Data shown as mean ± S.E.M. ns, not significant, by unpaired 2-sided t-test. Source data are provided as a Source Data file.

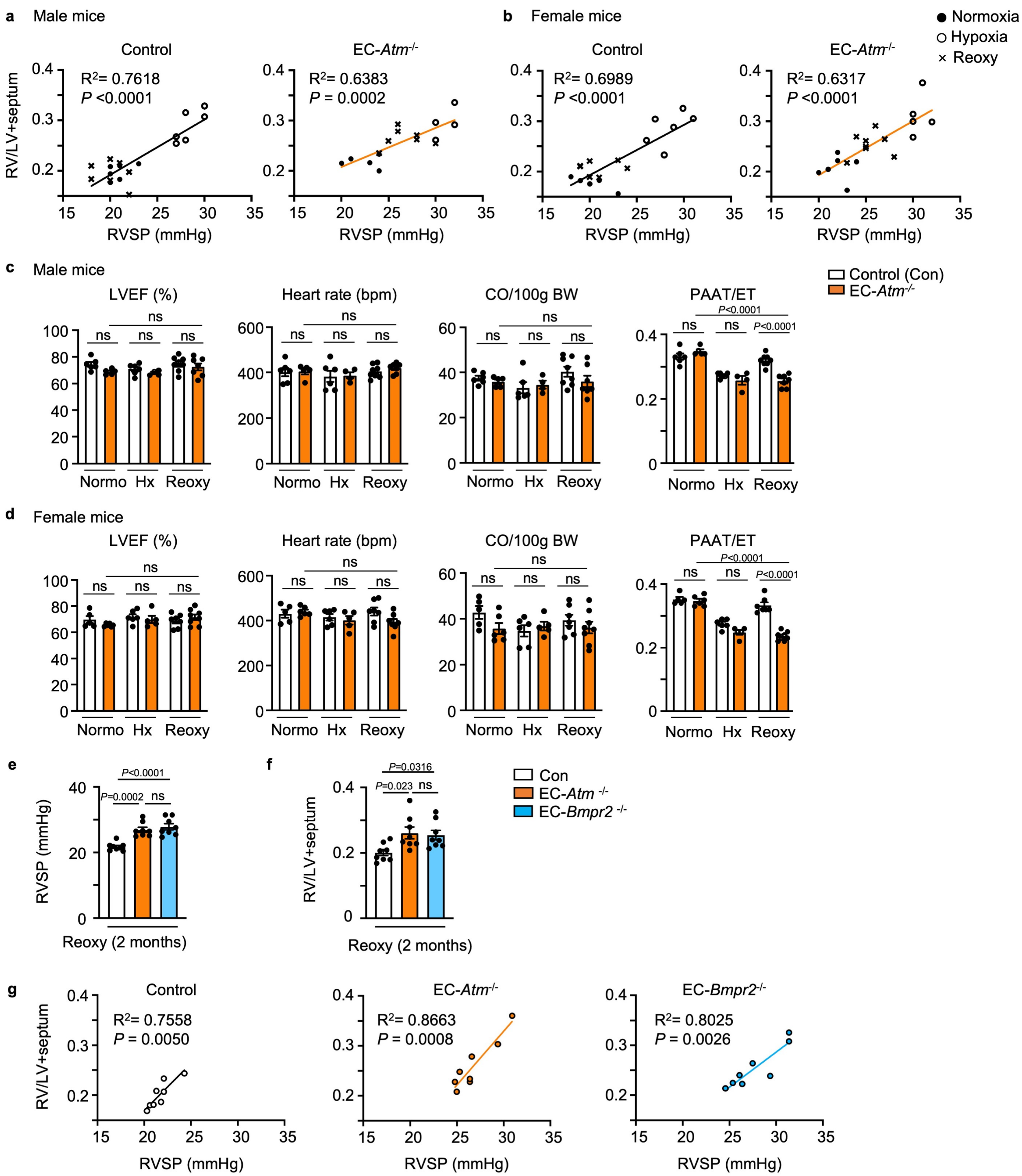

Supplementary Figure 3: Additional characterization of EC-*Atm*$^{-/-}$ mice

a, b, Scatter plot showing a correlation between right ventricular systolic pressure (RVSP) and RV hypertrophy (RV/LV+septum) shown in Figure 3a and b.

(c, d) Left ventricular ejection fraction (LVEF), heart rate, cardiac output per 100g body weight (CO/100g BW), and pulmonary artery acceleration time per ejection time (PAAT/ET) in **(c)** male (Control: Normo n=6, Hx n=6, Reoxy n=8; EC-*Atm*$^{-/-}$: Normo n=5, Hx n=4, Reoxy n=7) and **(d)** female (Control: Normo n=5, Hx n=6, Reoxy n=7; EC-*Atm*$^{-/-}$: Normo n=6, Hx n=5, Reoxy n=8) Control or EC-*Atm*$^{-/-}$ mice. Each data point represents a mouse. Bars represent mean ± S.E.M. P values determined by 2-way ANOVA with Holm-Sidak posthoc test. ns, not significant. (**e, f)** RVSP and RV/LV+septum in male Control, EC-*Atm*$^{-/-}$ or EC-*Bmpr2*$^{-/-}$ mice, after two months of reoxygenation following hypoxia**.** Each data point represents a mouse. n = 8 mice per condition. Bars represent mean ± S.E.M. P values determined by one-way ANOVA with Holm-Sidak posthoc test. **g,** Scatter plot showing a correlation between right ventricular systolic pressure (RVSP) and RV hypertrophy (RV/LV+septum) in all three groups of mice. Source data are provided as a Source Data file.

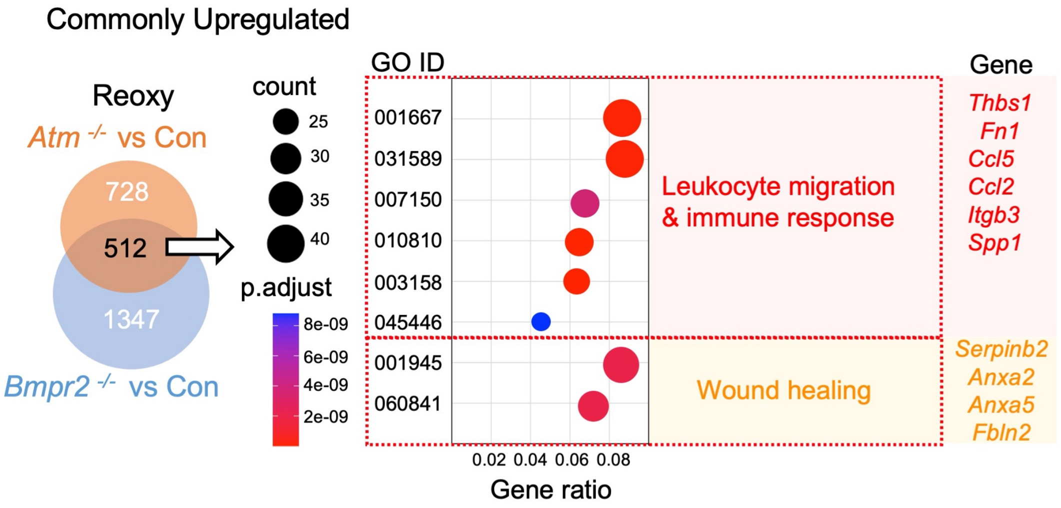

Supplementary Figure 4: Transcriptome analysis reveals inflammatory pathways commonly upregulated in *Bmpr2* and *Atm* deleted murine PAEC.

Venn diagram showing the number of genes upregulated in lung EC of EC-*Atm*$^{-/-}$ or EC-*Bmpr2*$^{-/-}$ under Reoxy, compared to control mice [adjusted p-value (padj) <0.05]. Gene Ontology (GO) analysis was performed for 512 commonly upregulated genes and showed Top GO pathways. P-values were adjusted using the Benjamini-Hochberg method for multiple comparison by one-sided Fisher's exact test.

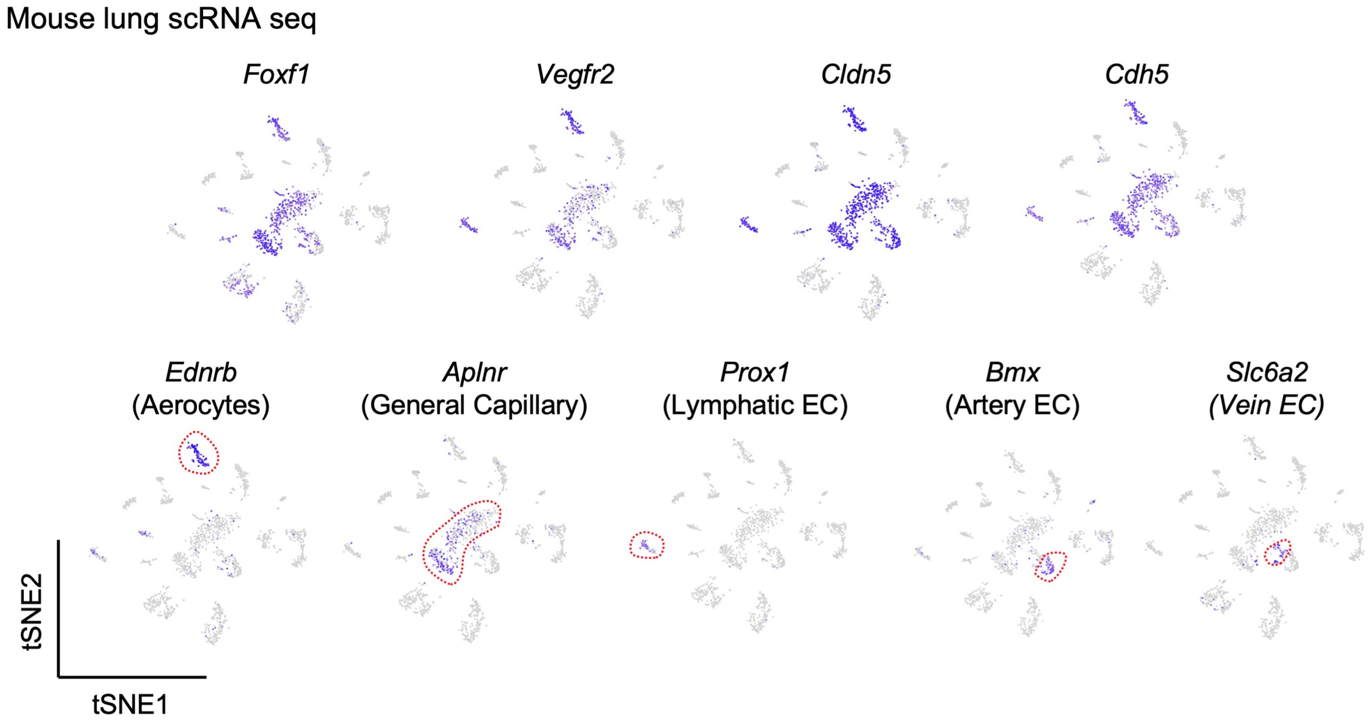

Supplementary Figure 5: Mouse lung scRNA seq data from public data.

Mouse lung single-cell (sc)RNA seq data were obtained from publicly available link（https://tabula-muris.ds.czbiohub.org）. *Ednrb* (aerocyte), *Aplnr* (general capillary), *Prox1* (lymphatic EC), *Bmx* (artery EC), *Slc6a2* (vein EC) were used to distinguish mouse endothelial cell types[3, 4]. *Foxf1*, *Vegfr2*, *Cldn5* and *Cdh5* were expressed in lung endothelial cell types. *Foxf1* was highly expressed in lung endothelial cells except lymphatic EC, but minimally expressed in some non-EC cells (stromal cells).

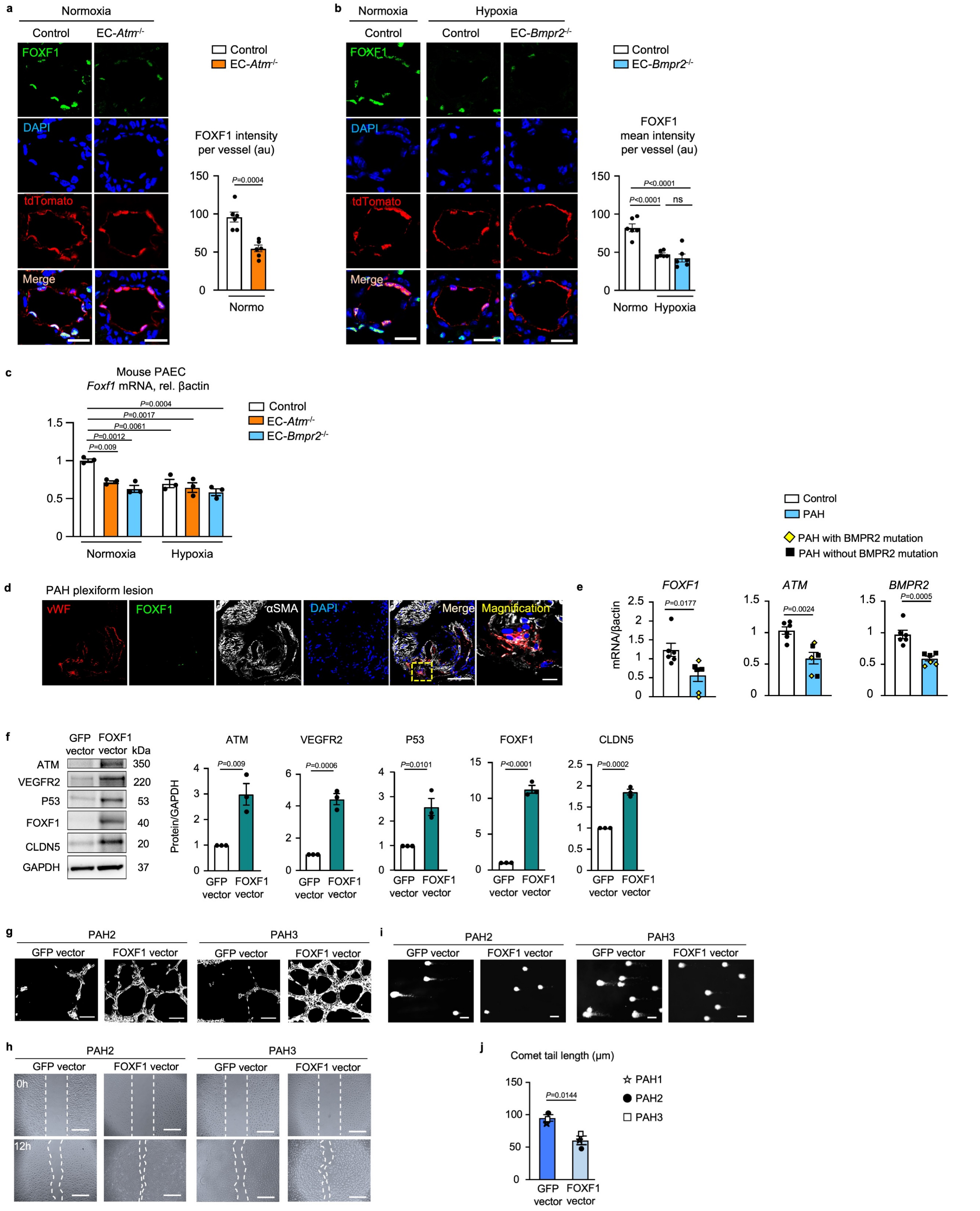

Supplementary Figure 6: FOXF1 is linked to angiogenesis and DNA damage repair in PAEC. *(See legend on the next page)*

Supplementary Figure 6: FOXF1 is linked to angiogenesis and DNA damage repair in PAEC.

(a, b) FOXF1 immunohistochemistry of pulmonary artery in (a) control or EC-*Atm*$^{-/-}$ mice under normoxia, and (b) EC-*Bmpr2*$^{-/-}$ mice under hypoxia or control mice under normoxia or hypoxia. The mean FOXF1 intensity was quantified of 5 vessels per mouse in n=6 mice in each group. Scale bar, 50 µm.

c, *Foxf1* expression in lung EC of EC-*Atm*$^{-/-}$, EC-*Bmpr2*$^{-/-}$ and control mice in normoxia or hypoxia.

d. Immunohistochemistry for FOXF1 and the EC marker von Willebrand factor (vWF) in plexiform lesion in lung sections of PAH patients. Few endothelial cells have detectable FOXF1 in plexiform lesion. Scale bar, 50 µm. The panel on the far right shows a magnified merged image of the area delineated by the dotted line. Scale bar, 10 µm.

e, mRNA expression of *FOXF1, ATM* and *BMPR2* in cultured PAEC from PAH patients (3 patients with a BMPR2 mutation and 3 patients without a BMPR2 mutation) or 6 healthy donor controls. Bars represent mean ± S.E.M. P values determined by unpaired 2-sided t-test.

f. Immunoblot and densitometry of ATM, VEGFR2, P53 FOXF1 and CLDN5 in human PAEC after overexpression of FOXF1 gene (FOXF1 vector) or GFP (GFP vector) by transfection with lentivirus vector. Bars represent mean ± S.E.M. P values determined by unpaired 2-sided t-test.

(g-j). Angiogenesis function and DNA damage after FOXF1 overexpression. FOXF1 was overexpressed in PAEC of PAH patients by transfection with lentivirus vector carrying the *FOXF1* gene (FOXF1 vector*)* or GFP as control (GFP vector). Images of PAH1 patient

and quantitation of the data from the three PAH patients is shown in Figure 5 of the Main text. **g.** Representative images of tube formation of PAEC of PAH patients PAH2 and PAH3. Scale bar, 200 µm. **h.** Representative images of scratch assay of PAH patients PAH2 and PAH3 at 0 and 12 h. Scale bar, 450 µm. **i.** Representative images of comet assay of PAH patients PAH2 and PAH3. Scale bar, 20 μm. **j,** mean comet tail length of PAH patients PAH1, PAH2 and PAH3. Bars represent mean ± S.E.M. P values determined by unpaired 2-sided t-test. Source data are provided as a Source Data file.

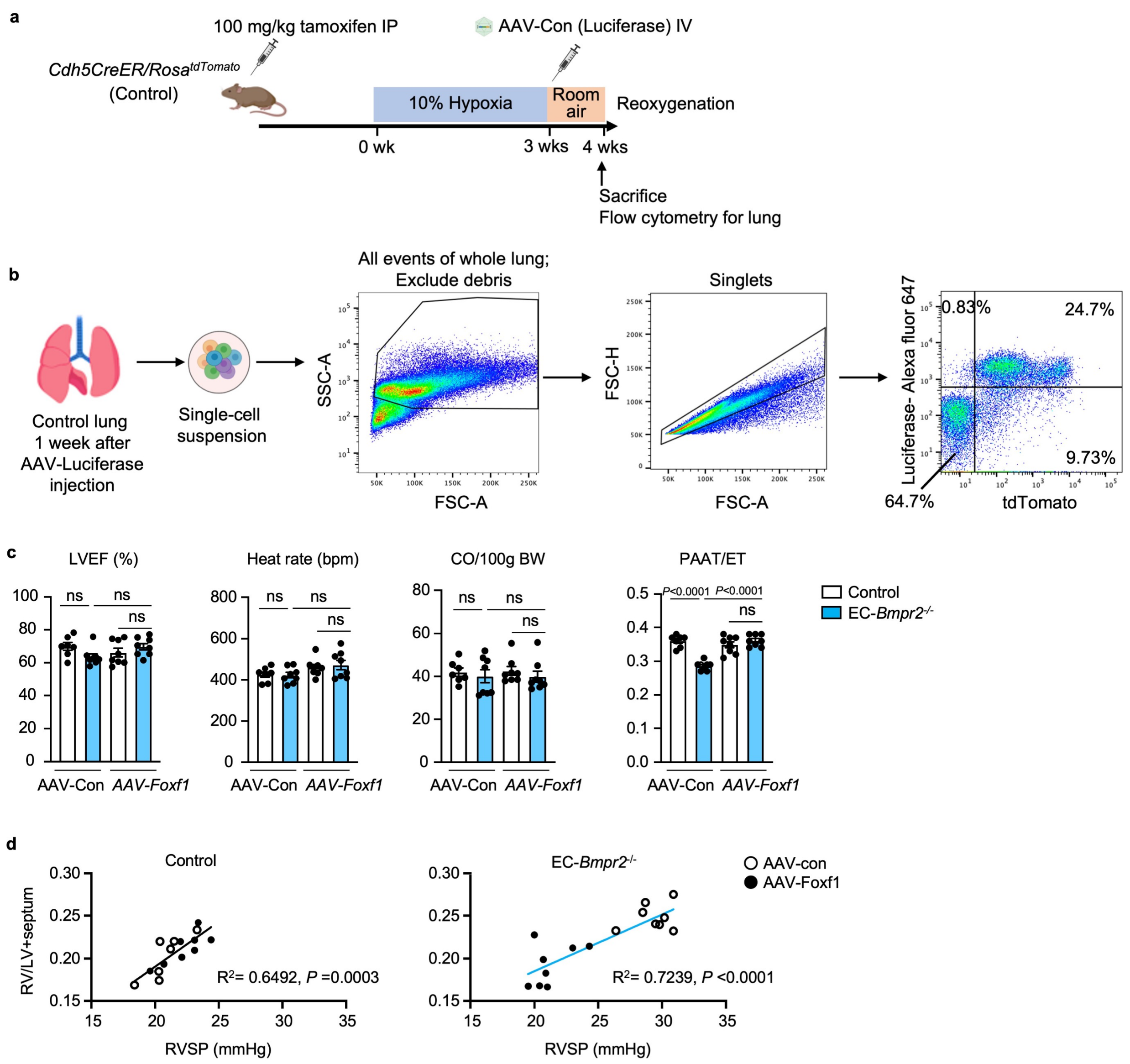

Supplementary Figure 7: Cardiac function of EC-*Bmpr2*$^{-/-}$ or control mice exposed to the reoxygenation protocol following *Foxf1* delivery to the lung EC.

a. Schema of experimental design: *Cdh5CreER/Rosa*^{tdTomato} (control) mice were exposed to hypoxia (10% oxygen) for 3 weeks after tamoxifen injection, transferred to room air and administered AAV-luciferase, and sacrificed a week after AAV-luciferase administration. Schema created with BioRender.com. **b.** Mouse lungs were digested enzymatically, and flow cytometry was applied to the single cell suspension of the whole lung. Schema

created with BioRender.com. **c.** AAV-*Foxf1* or AAV-luciferase (AAV-Con) as control were administered via tail vein injection to male EC-*Bmpr2*$^{-/-}$ or control mice following 3 weeks in hypoxia (10% O_2). The mice were returned to room air for 4 weeks. Left ventricular ejection fraction (LVEF), heart rate, cardiac output per 100g body weight (CO/100g BW), and Pulmonary artery acceleration time per ejection time (PAAT/ET) were measured after reoxygenation for 4 weeks. Control mice with AAV-Con, n=7; EC-*Bmpr2*$^{-/-}$ mice with AAV-Con, control mice with AAV-*Foxf1*, and EC-*Bmpr2*$^{-/-}$ mice with AAV-*Foxf1*, n=8. Each dot represents a mouse. Bars represent mean ± S.E.M. P values determined by 2-way ANOVA with Holm-Sidak posthoc test. ns, not significant.

d, Scatter plot showing a correlation between right ventricular systolic pressure (RVSP) and RV hypertrophy (RV/LV+septum) shown in Figure 7c. Source data are provided as a Source Data file.

Supplementary References

1. Kurtz, D.M. & Travlos, G.S. The clinical chemistry of laboratory animals. (CRC Press is an imprint of the Taylor & Francis Group, an Informa business, Boca Raton, FL, 2018).
2. Zhou, X. & Hansson, G.K. Effect of sex and age on serum biochemical reference ranges in C57BL/6J mice. *Comp Med* **54**, 176-178 (2004).
3. Gillich A., Zhang F., Farmer C.G., Travaglini K.J., Tan S.Y., Gu M., Zhou B., Feinstein J.A., Krasnow M.A. & Metzger R.J. Capillary cell-type specialization in the alveolus. *Nature.* **586,** 785-789 (2020)
4. Travaglini K.J., Nabhan A.N., Penland L., Sinha R., Gillich A., Sit R.V., Chang S., Conley S.D., Mori Y., Seita J., Berry G.J., Shrager J.B., Metzger R.J., Kuo C.S., Neff N., Weissman I.L., Quake S.R., Krasnow M.A. A molecular cell atlas of the human lung from single-cell RNA sequencing. *Nature* **587**, 619-625 (2020).
