# Supplementary material for: Reduced FOXF1 links unrepaired DNA damage to pulmonary arterial hypertension
Source: Nat Commun. 2023 Nov 21;14:7578. doi: 10.1038/s41467-023-43039-y (PMC10663616; doi:10.1038/s41467-023-43039-y)
Supplement: Supplementary file 3 — Reporting Summary [file 41467_2023_43039_MOESM3_ESM.pdf]

nature portfolio

Corresponding author(s): Marlene Rabinovitch, MD

Last updated by author(s): Oct 20, 2023

Reporting Summary

Nature Portfolio wishes to improve the reproducibility of the work that we publish. This form provides structure for consistency and transparency in reporting. For further information on Nature Portfolio policies, see our Editorial Policies and the Editorial Policy Checklist.

Statistics

For all statistical analyses, confirm that the following items are present in the figure legend, table legend, main text, or Methods section.

n/a	Confirmed	
☐	☒	The exact sample size (*n*) for each experimental group/condition, given as a discrete number and unit of measurement
☐	☒	A statement on whether measurements were taken from distinct samples or whether the same sample was measured repeatedly
☐	☒	The statistical test(s) used AND whether they are one- or two-sided<br>*Only common tests should be described solely by name; describe more complex techniques in the Methods section.*
☐	☒	A description of all covariates tested
☐	☒	A description of any assumptions or corrections, such as tests of normality and adjustment for multiple comparisons
☐	☒	A full description of the statistical parameters including central tendency (e.g. means) or other basic estimates (e.g. regression coefficient) AND variation (e.g. standard deviation) or associated estimates of uncertainty (e.g. confidence intervals)
☐	☒	For null hypothesis testing, the test statistic (e.g. *F*, *t*, *r*) with confidence intervals, effect sizes, degrees of freedom and *P* value noted<br>*Give P values as exact values whenever suitable.*
☒	☐	For Bayesian analysis, information on the choice of priors and Markov chain Monte Carlo settings
☒	☐	For hierarchical and complex designs, identification of the appropriate level for tests and full reporting of outcomes
☒	☐	Estimates of effect sizes (e.g. Cohen's *d*, Pearson's *r*), indicating how they were calculated

Our web collection on statistics for biologists contains articles on many of the points above.

Software and code

Policy information about availability of computer code

Data collection	For bulk RNA-seq, Base call files from Illumina sequencing were converted to fastq format using Bcl2fastq.<br>For single-cell RNA-seq, 10x sequence de-multiplexing, alignment, and UMI counting: CellRanger v6.0.0.
Data analysis	For RNA-Seq analysis, the FASTQ reads were QC-checked using FastQC v0.11.9 and underwent quality and adapter trimming using Trim Galore v0.6.6. The resulting FASTQ reads were analyzed using the nf-core/rnaseq pipeline v3.3 (https://nf-co.re/rnaseq) and aligned to the mouse reference genome mm10 using STAR v2.6.1d. The aligned transcripts were quantitated by RSEM v1.3.1 (http://deweylab.github.io/RSEM/).<br>Differentially expressed genes were detected using DESeq2 v 1.32.0. GO enrichment analysis was performed using clusterProfiler v4.4.1.<br>For single-cell RNA-seq: Filtering, clustering, and annotating cells: Seurat v3.0.2, R v3.6.3.<br>Images were analyzed using Zeiss ZEN software blue version (version 3.4) and ImageJ (version 2.1.0).<br>Flow cytometry data were analyzed by Flowjo software (version 10.7.1).<br>Other data was analyzed by Prism (version 9.3.1).

For manuscripts utilizing custom algorithms or software that are central to the research but not yet described in published literature, software must be made available to editors and reviewers. We strongly encourage code deposition in a community repository (e.g. GitHub). See the Nature Portfolio guidelines for submitting code & software for further information.

Data

Policy information about availability of data

All manuscripts must include a data availability statement. This statement should provide the following information, where applicable:

- Accession codes, unique identifiers, or web links for publicly available datasets
- A description of any restrictions on data availability
- For clinical datasets or third party data, please ensure that the statement adheres to our policy

Bulk RNA-Seq and single-cell RNA-seq data is available from the Gene Expression Omnibus (GEO), under accession number GSE215933 [https://www.ncbi.nlm.nih.gov/geo/query/acc.cgi?acc=GSE215933]. The potential Foxf1 target genes were provided by ChIP-Atlas database (https://chip-atlas.org/target_genes). Mouse lung scRNA seq data in supplementary figure 5 was provided by Tabula Muris (https://tabula-muris.ds.czbiohub.org).
All other data generated in this study and presented in figures are provided in the Supplementary "Source Data" file.

Research involving human participants, their data, or biological material

Policy information about studies with human participants or human data. See also policy information about sex, gender (identity/presentation), and sexual orientation and race, ethnicity and racism.

Reporting on sex and gender	The donor of primary human pulmonary artery endothelial cells (PAEC) commercially obtained from PromoCell (C-12241) was male. We used lung tissues and PAEC from Healthy donors (five males and four females), PAH patients (four male and ten females). Sex was provided by the procuring center and is reported in Online Supplement Table 3.<br>In the human PAH cells, individual patient analysis was carried out. The table relates the patient number to the patient gender. In the tissue samples, the data reflected the aggregate of both males and females in each group. As shown in the table, the BMPR2 mutant group comprised only females, the PAH non-BMPR2 mutant group included 2 males and 2 females and the control group included 2 males and 2 females. The numbers were too small to disaggregate the data for statistical analysis.
Reporting on race, ethnicity, or other socially relevant groupings	Race and ethnicity were not linked to the cell culture studies. Other than the BMPR2 mutation information, no further genomic information was provided for these samples.
Population characteristics	The studies conducted with human cells do not constitute Human Subject Research because the cell lines we used were provided to us coded with no identifying information. Banked cell lines were commercially obtained (PromoCell) or harvested from PAH patients' lungs obtained through the Pulmonary Hypertension Breakthrough Initiative (PHBI) as detailed in the Methods section. The relevant population characteristics are included in Supplementary Table 3.
Recruitment	Cells purchased from PromoCell were derived from tissues of donors who have signed an informed consent form. Human tissues provided by the PHBI Initiative were obtained under the PHBI network protocol, approved by the Medical School IRB at the University of Michigan, and written informed consent at the lung procurement sites. There was no compensation to the donors for participation in the study. The cell lines used were coded with no identifying information. The informed consent outlined in detail the purpose of the donation and the procedure for processing the tissue.
Ethics oversight	Cells purchased from PromoCell were derived from tissues of donors who have signed an informed consent form. Human tissues provided by the PHBI Initiative were obtained under the PHBI network protocol, approved by the Medical School IRB at the University of Michigan, and written informed consent at the lung procurement sites. There was no compensation to the donors for participation in the study. The cell lines used were coded with no identifying information. The informed consent outlined in detail the purpose of the donation and the procedure for processing the tissue.

Note that full information on the approval of the study protocol must also be provided in the manuscript.

Field-specific reporting

Please select the one below that is the best fit for your research. If you are not sure, read the appropriate sections before making your selection.

☒ Life sciences ☐ Behavioural & social sciences ☐ Ecological, evolutionary & environmental sciences

For a reference copy of the document with all sections, see nature.com/documents/nr-reporting-summary-flat.pdf

Life sciences study design

All studies must disclose on these points even when the disclosure is negative.

Sample size	Sample size calculations were performed and in keeping with our published studies (Diebold et al., 2015, manuscript ref. 18), Spiekerkoetter et al., 2013, manuscript ref. 21) and our experience with similar experimental designs. We chose n=8/group when expected variance is <30% and expected effect size >40% (power calculation >0.8); n=6/group is used when expected variance <20% and effect size >50%.<br><br>In the hypoxia groups, the male cohort consisted of n=6 male control and n=4 male EC-Atm-/- mice, and the female cohort consisted of n=6 control and n=5 EC-Atm-/- mice. We did not pursue larger cohorts because the emphasis was on the phenotype during reoxygenation, and we observed no differences in the DNA damage response during hypoxia. There was no attrition in our experiments and all mice were included in

the data analysis.

Data exclusions	No data were excluded from the study.
Replication	Numbers of experimental biological replicates are described in each figure legend. Experiments were performed multiple times to ensure that results are reproducible. All attempts at replication were successful.
Randomization	Male and female mice were randomly assigned into the experimental groups. For the studies of human cells, we randomly selected samples of PAH patients or controls 20-50 years of age for the study.
Blinding	Hemodynamic measurements in mice were not blinded during data collection and analysis. Experiments performed under different conditions (Hypoxia, normoxia, and reoxy) take place on different days because of feasibility. As well, the cages are labeled with the genotype so the investigator was not blinded to the genotype when handling EC-Atm-/- , EC-Bmpr2-/- and control mice.<br><br>Quantification of the images of the immunostaining and comet assays were performed in a blinded manner, i.e. the investigators that analyzed the images were not aware of the experimental conditions from which these images were taken.<br><br>The investigators were not blinded when performing the other cell culture experiments.

Reporting for specific materials, systems and methods

We require information from authors about some types of materials, experimental systems and methods used in many studies. Here, indicate whether each material, system or method listed is relevant to your study. If you are not sure if a list item applies to your research, read the appropriate section before selecting a response.

Materials & experimental systems

n/a	Involved in the study
☐	☒ Antibodies
☐	☒ Eukaryotic cell lines
☒	☐ Palaeontology and archaeology
☐	☒ Animals and other organisms
☒	☐ Clinical data
☒	☐ Dual use research of concern
☒	☐ Plants

Methods

n/a	Involved in the study
☒	☐ ChIP-seq
☐	☒ Flow cytometry
☒	☐ MRI-based neuroimaging

Antibodies

Antibodies used	For immunostaining, primary antibodies were used targeting aSMA (A2547, Sigma Aldrich, lot# 076M4784V, IF 1:300, IHC 1:400. ab5694, Abcam, lot# 449256, IF 1:50), MECA32 (AB_531797, Developmental Studies Hybridoma Bank, lot# 6/20/19-17ug/ml, IF 1:4), Luciferase (ab21176, Abcam, lot# GR3355137-1, IF 1:100), Von Willebrand factor (ab6994, Abcam, lot# GR275409-1, IF 1:300), RFP (600-401-379,Rockland, lot# 42872, IF 1:1000), tdTomato (TA150129, Origene, lot# 0316, IF 1:500), γH2AX (05-636, Millpore, lot# 2138016, IF 1:100, ICC 1:300) and Phospho-RPA(S4/S8) (A300-245A, Bethyl, ICC 1:500). Secondary antibodies were Alexa Fluorconjugated donkey anti-mouse Alexa Fluor 647 (A31571, lot# 1984047, IF, ICC 1:500) , donkey anti-rabbit Alexa Fluor 647 (A31573, lot# 1964354, IF, ICC 1:500, Flow cytometry 1:1000) , donkey anti-rabbit Alexa Fluor 594 (A21207, lot# 1890862, IF, ICC 1:500), donkey anti-goat Alexa Fluor 594 (A11058, lot# 2045324,IF, ICC 1:500), donkey anti-mouse Alexa Fluor 488 (A21202, lot# 1796361, IF, ICC 1:500), donkey anti-rabbit Alexa Fluor 488 (A21206, lot# 2045215, IF, ICC 1:500), donkey anti-goat Alexa Fluor 488 (A11055, lot# 1869589, IF, ICC 1:500); all from Thermo Fisher Scientific.<br><br>For western blotting, primary antibodies were used targeting BMPR2 (clone 18)(612292, BD bioscience, lot# 7299907/ 7341692, 1:250), Phospho-RPA(S4/S8) (A300-245A, Bethyl, lot# N/A, 1:1000), RPA (A300-244A, Bethyl, lot# N/A, 1:1000), γH2AX (Clone JBW 301) (05-636, Millpore, lot# 33380399, 1:1000), Bactin (clone C4) (sc-47778, Santa cruz, lot# J1113, 1:2000), tdTomato (TA150129, origene, lot# 0136, 1:5000), ATM (clone D2E2) (2873, Cell signaling, lot# 5, 1:1000), VEGFR2 (clone 55B11) (2479, Cell signaling, lot# 18, 1:1000), GAPDH (clone 14C10)(2118,Cell signaling, lot# 10, 1:1000), P53 (clone DO-1) (sc-126, Santa cruz, lot# J0113, 1:500), FOXF1 (AF4798, R and D, lot# AHH0420091, 1:1000), ab168383, Abcam, lot# GR3399493-4, 1:1000), CLDN5 (4C3C2, ThermoFisher, lot# 359666A, 1:500), DNA-PK (4602T, Cell signaling, lot# 2, 1:1000) and ATR (2790S, Cell signaling, lot# 10, 1:1000). Secondary antibodies were anti-rabbit antibodies (211-032-171, Jackson Immunoresearch, lot# 145158, 1:5000, sc-2004, Santa cruz, lot# B2216, 1:5000), anti-goat antibodies (sc-2953, Santa Cruz, lot# D2916, 1:5000) and anti-mouse antibodies (sc-2005, Santa cruz, lot# H2213, 1:5000).<br><br>For flow cytometry, Luciferase (Ab185923, Abcam, lot# GR331715-3, 1:200) was used followed by staining of donkey anti-rabbit Alexa Fluor 647 (A31573, Thermo Fisher Scientific, lot# 1964354, 1:1000).
Validation	All antibodies used in our study were validated by the respective commercial source for the application used.<br>aSMA (1A4): https://www.sigmaaldrich.com/US/en/product/sigma/a2547<br>aSMA: https://www.abcam.com/products/primary-antibodies/alpha-smooth-muscle-actin-antibody-ab5694.html<br>MECA32: https://dshb.biology.uiowa.edu/MECA-32<br>Luciferase: https://www.abcam.com/firefly-luciferase-antibody-ab21176.html<br>Luciferase: https://www.abcam.com/products/primary-antibodies/firefly-luciferase-antibody-epr17789-n-terminal-ab185923.html

vWF: https://www.abcam.com/von-willebrand-factor-antibody-ab6994.html
RFP: https://www.rockland.com/categories/primary-antibodies/rfp-antibody-pre-adsorbed-600-401-379/
tdTomato: https://www.origene.com/catalog/antibodies/tag-antibodies/ta150129/goat-polyclonal-tdtomato-antibody
γH2AX: https://www.emdmillipore.com/US/en/product/Anti-phospho-Histone-H2A.X-Ser139-Antibody-clone-JBW301,MM_NF-05-636
Phospho-RPA(S4/S8): https://www.fortislife.com/products/primary-antibodies/rabbit-anti-phospho-rpa32-s4-s8-antibody BETHYLA300-245
RPA: https://www.fortislife.com/products/primary-antibodies/rabbit-anti-rpa32-antibody/BETHYL-A300-244
BMPR2: https://www.bdbiosciences.com/en-fr/products/reagents/western-blotting-and-molecular-reagents/western-blot-reagents/Purified-Mouse-Anti-BMPR-II.612292
Bactin (C4): https://www.scbt.com/p/beta-actin-antibody-c4
ATM: https://www.cellsignal.com/products/primary-antibodies/atm-d2e2-rabbit-mab/2873
VEGFR2: https://www.cellsignal.com/products/primary-antibodies/vegf-receptor-2-55b11-rabbit-mab/2479
GAPDH: https://www.cellsignal.com/products/primary-antibodies/gapdh-14c10-rabbit-mab/2118
P53 (DO-1): https://www.scbt.com/p/p53-antibody-do-1
FOXF1: https://www.rndsystems.com/products/human-mouse-foxf1-antibody_af4798
FOXF1: https://www.abcam.com/foxf1-antibody-epr7971-ab168383.html
CLDN5: https://www.thermofisher.com/antibody/product/Claudin-5-Antibody-clone-4C3C2-Monoclonal/35-2500
DNA-PK: https://www.cellsignal.com/products/primary-antibodies/dna-pkcs-antibody/4602
ATR: https://www.cellsignal.com/products/primary-antibodies/atr-antibody/2790
Donkey anti-mouse Alexa Fluor 647: https://www.thermofisher.com/antibody/product/Donkey-anti-Mouse-IgG-H-L-Highly-Cross-Adsorbed-Secondary-Antibody-Polyclonal/A-31571
Donkey anti-rabbit Alexa Fluor 647: https://www.thermofisher.com/antibody/product/Donkey-anti-Rabbit-IgG-H-L-Highly-Cross-Adsorbed-Secondary-Antibody-Polyclonal/A-31573
Donkey anti-rabbit Alexa Fluor 594: https://www.thermofisher.com/antibody/product/Donkey-anti-Rabbit-IgG-H-L-Highly-Cross-Adsorbed-Secondary-Antibody-Polyclonal/A-21207
Donkey anti-goat Alexa Fluor 594: https://www.thermofisher.com/antibody/product/Donkey-anti-Goat-IgG-H-L-Cross-Adsorbed-Secondary-Antibody-Polyclonal/A-11058
Donkey anti-mouse Alexa Fluor 488: https://www.thermofisher.com/antibody/product/Donkey-anti-Mouse-IgG-H-L-Highly-Cross-Adsorbed-Secondary-Antibody-Polyclonal/A-21202
Donkey anti-rabbit Alexa Fluor 488: https://www.thermofisher.com/antibody/product/Donkey-anti-Rabbit-IgG-H-L-Highly-Cross-Adsorbed-Secondary-Antibody-Polyclonal/A-21206
Donkey anti-goat Alexa Fluor 488: https://www.thermofisher.com/antibody/product/Donkey-anti-Goat-IgG-H-L-Cross-Adsorbed-Secondary-Antibody-Polyclonal/A-11055
Anti-mouse horseradish peroxidase (HRP) antibody: https://datasheets.scbt.com/sc-2005.pdf
Anti-goat horseradish peroxidase (HRP) antibody: https://datasheets.scbt.com/sc-2953.pdf
Anti-rabbit horseradish peroxidase (HRP) antibody: https://datasheets.scbt.com/sc-2004.pdf
Anti-rabbit horseradish peroxidase (HRP) antibody: https://www.jacksonimmuno.com/catalog/products/211-032-171

Eukaryotic cell lines

Policy information about cell lines and Sex and Gender in Research

Cell line source(s)	Primary human pulmonary artery endothelial cells (PAEC) were either commercially obtained (C-12241, PromoCell) or harvested from PAH patients lungs obtained though the Pulmonary Hypertension Breakthrough Initiative (PHBI), funded by the NIH (R24 HL123767) and the Cardiovascular Medical Research and Education Fund (CMREF; UL 1RR024986).
Authentication	Cell lines were routinely checked for endothelial marker expression.
Mycoplasma contamination	Cells were routinely tested for mycoplasma contamination.
Commonly misidentified lines (See ICLAC register)	N/A

Animals and other research organisms

Policy information about studies involving animals; ARRIVE guidelines recommended for reporting animal research, and Sex and Gender in Research

Laboratory animals	The following mouse strains were used:<br>Rosa tdTomato Cre reporter (The Jackson Laboratory, B6.Cg Gt(ROSA)26Sortm14(CAGtdTomato) Hze/J, Stock # 007914).<br>Cdh5CreER (kindly obtained from Dr. Kristy Red-Horse).<br>Atm fl/fl (The Jackson Laboratory, 129-Atmtm2.1Fwa/J, Stock # 021444).<br>Bmpr2fl/fl mice (described in our lab as previously).<br>Cdh5CreER mice were bred with Rosa tdTomato Cre reporter mice and used as control mice.<br>Atmfl/fl or Bmpr2fl/fl mice were crossed with Cdh5CreER/Rosa tdTomato mice.<br><br>Mice were ranged in age from 7-10 weeks at the start of the experiment. Mice were housed under standard conditions according to protocols of the Stanford University Institutional Animal Care and Use Committees in pathogen-free, individually ventilated microisolator cages in a room with a 12 hours light/dark cycle, and a temperature- and humidity controlled environment of 20-26°C and 30-70% humidity, with access to food and water ad libitum.
Wild animals	The study did not involve wild animals.

Reporting on sex	EC- Atm-/- mice that underwent the haemodynamics study in Figure 3 (a and b) and Supplemental Figure 3 (c and d) were males and females.<br>EC-Bmpr2-/- mice studied by immunofluorescence, RNA-seq and AAV treatment were males.<br>EC- Atm-/- mice studied by immunofluorescence and RNA-seq were males.<br>Control mice were sex-matched to transgenic mice in each experiment.
Field-collected samples	The study did not involve samples from the field.
Ethics oversight	The Animal Care Committee at Stanford University approved all the experimental protocols used in this study under Stanford APLAC protocol 10704, in accordance with the guidelines of the American Physiological Society.

Note that full information on the approval of the study protocol must also be provided in the manuscript.

Flow Cytometry

Plots

Confirm that:

☒ The axis labels state the marker and fluorochrome used (e.g. CD4-FITC).

☒ The axis scales are clearly visible. Include numbers along axes only for bottom left plot of group (a 'group' is an analysis of identical markers).

☒ All plots are contour plots with outliers or pseudocolor plots.

☒ A numerical value for number of cells or percentage (with statistics) is provided.

Methodology

Sample preparation	For the murine whole lung experiment, control mice (Cdh5CreER/RosatdTomato) injected with tamoxifen were treated with AAV-luciferase after 3 weeks of hypoxia (10% O2). Lungs were harvested one week after AAV injection and digested enzymatically. A single-cell suspension was prepared by passing the digested tissue through a 70 µm nylon strainer. Red blood cells were depleted in RBC lysis buffer (eBioscience # 00-4333-57) on ice for 2 mins, washed with cold PBS, and centrifuged at 300 g for 5 mins. Single cell suspensions were incubated with TruStain FcX (Biolegend #101319) and stained with fixable viability dye (Biolegend # 423101) followed by fixation and permeabilization (BD # 554714) according to the supplier instructions. The cells were stained with luciferase antibody (1:200Abcam #Ab185923) at room temperature for 30 min followed by staining of Alexa fluor 647.<br><br>For the ROS experiment, PAEC were transfected with siRNA and cultured in a 6-cm dish in the hypoxia chamber (0.5% oxygen) for 48 hours. For the assay, hypoxia samples were incubated in normal ECM medium containing 2.5 µM CMH2DCFDA (C6827; ThermoFisher) for 15 min at 37°C. For samples of reoxygenation (15 min, 30 min and 60 min), the cells were transferred to normoxia and incubated in normal ECM medium containing 2.5µM CM-H2DCFDA). The cells were washed twice with PBS and then trypsinized. Fluorescence (excitation and emission wavelengths of 498 and 522 nm, respectively) was measured by flow cytometry.
Instrument	Data were collected by BD FACS Aria Fusion or BD FACS Aria II.
Software	FlowJo v10.7.1.
Cell population abundance	We did not sort cells in this experiment.
Gating strategy	The Single cell suspension from murine whole lung was gated based on FSC/SSC properties followed by exclusion of debris and doublets. Then, dead cells were excluded from analysis. The gating strategy is described in supplementary figure and figure legend.

☒ Tick this box to confirm that a figure exemplifying the gating strategy is provided in the Supplementary Information.
